# The Influence of Social Media on the Treatment of Acne in Saudi Arabia

**DOI:** 10.7759/cureus.23169

**Published:** 2022-03-15

**Authors:** Reem K Bahaj, Zahraa H Alsaggaf, Mohammed H Abduljabbar, Jehad O Hariri

**Affiliations:** 1 Department of Dermatology, King Abdulaziz University Faculty of Medicine, Jeddah, SAU; 2 Department of Dermatology, King Abdulaziz University Hospital, Jeddah, SAU

**Keywords:** education, recommendation, treatment, social media, acne

## Abstract

Objectives

The purpose of this study was to examine the effects of social media on acne treatment among the Saudi Arabian population.

Methods

This was a cross-sectional survey-based study conducted from January 2021 to August 2021. A self-administered survey was distributed through social media to different regions of Saudi Arabia. The survey obtained participants’ sociodemographic information and details on whether people used social media for advice on acne treatment. It also asked whether they noticed any change in their acne based on social media recommendations.

Results

Of the 5,539 respondents, 4,227 experienced acne, of which 1,793 were influenced by social media. Most respondents were women. The majority of social media users were between 18 and 25 years old and chose social media as their first approach for acne advice. The most commonly used platform was Instagram (34%). The most frequent social media recommendation chosen was to increase water intake. Many noticed a slight change in their acne (64%), and 14.9% had side effects. There was a significant association between the use of social media for advice and gender (p-value < 0.001), education level (p-value = 0.002), and severity of acne (p-value < 0.001).

Conclusion

Social media has an influence on acne treatment, with many advice not recommended by established guidelines. These findings imply that dermatologists should pinpoint inaccuracies resulting from advice found on social media.

## Introduction

The Internet has made possible new forms of social interaction and information transmission within just two decades [1]. In 2017, an estimated 3.58 billion individuals had Internet access, and about 80% of Internet users looked up medical information online. While the Internet and social media have transformed how we communicate and interact with each other, they have also revolutionized how medical information becomes available, is compiled, and is distributed among the consumers at large. Thus, social media has become a remarkable source of knowledge for people, including those suffering from skin diseases [2,3].

Dermatology, or “skincare,” as it is usually referred to on social media platforms, has become a popular topic that is increasingly acquiring public attention and recognition [4]. This popularity is thought to be due to the idea of flawless skin displayed on social media networks, establishing clear and spotless skin as the norm. Meanwhile, the idea of accepting less-than-perfect skin is being overlooked, with individuals who suffer from flawed or troubled skin facing stigmatization. According to a study on skin disease and its relationship with contemporary societal pressures, the pursuit of youthful beauty is now believed to be a universal feature of life [5].

Acne is a chronic inflammatory disease characterized by multifactorial lesions, ranging from comedones to papules, pustules, and nodules. It normally begins in adolescence, regresses in the mid-20s, and may be more frequent and severe in men. Approximately 85% of adolescents and young adults suffer from acne [6]. Therefore, acne vulgaris can have major psychological and social consequences for individuals dealing with it, because it can last for years and leave permanent scars on the skin despite treatment [7,8].

The psychological impact of skin issues is primarily attributable to the appearance of the condition rather than its symptoms. Earlier studies showed that while the pain and pruritus accompanying acne were occasionally bothersome for some people, the majority, particularly women, believed that their appearance-related problems stemmed from the creation of a social ideal of perfect skin. As a result, it is widely acknowledged that social media can affect an individual’s decision to seek medical treatment [9]. The fact that acne can have a long-term course and bring about visible skin issues has a severe impact on an individual’s psychosocial functioning and quality of life, forcing them to search for treatment [10].

According to a study conducted in West Virginia, 45% of participants used social media as a hub for acne treatment recommendations, and women accounted for about 75% of this population. Furthermore, the study found that YouTube and Instagram were the most popular platforms (both at 58%), with 81% of social media users having tried an over-the-counter product [11]. Another study in Turkey found that the most commonly utilized social media networks were Facebook (81%), Instagram (82.6%), and YouTube (66.1%). The interviewed patients exhibited a high level of interest in the content they discovered through social and conventional media on acne conditions. Of the patients, 51.2% only paid attention to the information, and 55.4% applied the learned information to practice [12].

The literature suggests that social media has a significant impact on acne therapy. There are limited studies conducted in Saudi Arabia that focus on the influence of social media on acne treatment; therefore, the purpose of this study was to assess how social media influences acne treatment while also determining the most common initial techniques used for acne management among the Saudi Arabian population.

## Materials and methods

Survey methods and questionnaire content

This cross-sectional study was approved by the Research Ethics Committee of King Abdulaziz University (reference number 32-21). This study was conducted from January 2021 to August 2021. All participants provided their informed consent while being notified of the objectives and confidentiality of this study.

The online survey was self-administered and was distributed throughout different social media platforms. It involved a representative sample of individuals of both genders from different regions of Saudi Arabia who had suffered from acne before, were at least 13 years old, and had access to social media. Respondents who did not have acne vulgaris were excluded from the study. The sample size was calculated using a 95% confidence interval (CI) and a 5% error margin. The survey was created using Google Forms (Appendix).

The questionnaire included aspects about the participants’ demographics, presence of acne, self-assessed acne severity, their first approach to acne treatment, social media as an acne treatment guide, and social media treatment suggestion that they have used, including over-the-counter products, self-made products, dietary changes, changes in physical activity, and improvements, if any, in their acne after using social media recommendations.

This survey did not collect any identifying information of the participants in order to ensure anonymity.

Data entry and analysis

Completed questionnaires were entered into a secure Excel sheet. Data were analyzed using Statistical Package for the Social Sciences (SPSS) version 24 (IBM Corp., Armonk, NY, USA). The respondents were categorized into two groups: those who used social media and those who did not, in response to the question “Have you used an acne treatment based on a social media suggestion?” Descriptive frequencies were used to interpret demographics and survey responses. The differences between social media users and nonusers based on gender, age, acne severity, and the first treatment approach were assessed among other variables. Chi-square and Fisher’s exact tests were used to assess the relationship between social media usage and gender, education, and acne severity.

## Results

This study aimed to assess the effect of social media on acne treatment. Overall, 5,539 responses were collected in this study, of which 4,227 (76.3%) consisted of participants having experienced acne. Those who had never suffered from acne constituted 1,312 (23.7%) of the responses and were therefore excluded from the analysis. Respondents who had had acne before were divided into those who had consulted social media for acne treatment and those who had not. Overall, most of the respondents were women (75.9%), were aged between 18 and 25 years (66%), were university-level graduates (72.6%), and had occasional acne (56.3%). Furthermore, most of the participants reported that their acne severity was moderate (52.1%). Table 1 presents the additional characteristics of the surveyed sample.

**Table 1:** Characteristics of the respondents * Percentage applies to row † Percentage applies to column

Characteristics		Consulted social media		Did not consult social media		Total		p-value
		Number	%^*^	Number	%^*^	Number	%^†^	
Gender	Female	1,572	49	1,636	51	3,208	75.9	<0.001
	Male	221	21.7	798	78.3	1,019	24.1	
Age group	Up to 17 years old	105	48.2	113	51.8	218	5.2	
	18–25 years old	1,281	45.9	1,510	54.1	2,791	66	
	26–35 years old	271	36.1	479	63.9	750	17.7	
	36–45 years old	99	28.9	243	71.1	342	8.1	
	46 and above years old	37	29.4	89	70.6	126	3	
Education level	Not educated	4	36.4	7	63.6	11	0.3	0.002
	Elementary graduate	13	31.7	28	68.3	41	1	
	Middle school graduate	66	42.3	90	57.7	156	3.7	
	High school graduate	351	42.2	481	57.8	832	19.7	
	College student or graduate	1329	43.3	1,739	56.7	3,068	72.6	
	Master’s degree and above	30	25.2	89	74.8	119	2.8	
Patient-reported current acne status	Yes	448	52	414	48	862	20.4	
	Sometimes	1,072	45	1,309	55	2,381	56.3	
	No	273	27.7	711	72.3	984	23.3	
Patient-reported acne severity	Light	497	32.6	1,028	67.4	1,525	36.1	<0.001
	Moderate	1,070	48.6	1,132	51.4	2,202	52.1	
	Severe	226	45.2	274	54.8	500	11.8	
Acne treatment first approach	Social media	449	89.1	55	10.9	504	11.9	
	Family or friends	268	45.3	323	54.7	591	14	
	Doctor	364	31.6	787	68.4	1,151	27.2	
	Pharmacist	197	37.7	326	62.3	523	12.4	
	Web search	382	69.1	171	30.9	553	13.1	
	Leave it as it is	129	14.5	759	85.5	888	21	
	Other	4	23.5	13	76.5	17	0.4	

Among all the respondents, 1,793 (42.4%) used social media for acne treatment recommendations, and they were mostly women (87.7%). There was a significant association between gender and the use of social media for advice (p < 0.001). When examining the relationship between education level and using social media for advice, it was found that 74.1% of those who used social media had a college-level education and that there was a positive association between the two variables (p = 0.002). In addition, there was a significant relationship between the severity of acne and turning to social media for acne treatment guidance (p < 0.001). Most respondents who used social media chose social media as their first approach for acne treatment advice (25%).

The most frequently used platform among social media users was Instagram (34%), followed closely by YouTube (33.2%). Furthermore, the most common advice users encountered on social media was to increase water intake (40.4%) and modify their diet (37.3%), as shown in Table 2. The majority of respondents found little change in their acne (64%), followed by no change at all (14.7%), and finally, only 21.2% found a marked change. Out of the social media users, 14.9% suffered side effects from the recommendations they found through social media.

**Table 2:** Characteristics of social media users ‡ Respondents can choose multiple answers

Characteristics	Number (%)	
Social media platform	5,468^‡^	
YouTube	1,207	33.2
Instagram	1,255	34
Snapchat	1,052	30.2
TikTok	610	20
WhatsApp	296	10.8
Facebook	69	2.8
Twitter	979	28.7
Acne treatment advice	7,479^‡^	
Rx without prescription (OTC)	1,236	33.7
Self-made product	1,007	29.3
Diet modification	1,445	37.3
Exercise modification	1,123	31.6
Supplements	1,016	29.4
Increase water intake	1,652	40.4

On the other hand, 57.6% of the respondents did not use social media for advice. Most opted to consult a doctor (32.3%) as their first approach or leave their acne untreated (31.2%).

## Discussion

Social media has become an increasingly popular source of health information for all individuals. Patients may be influenced by social media messages to purchase unsafe medications or try advertised treatments without consulting a doctor [13,14]. In this study, we aimed to characterize the effects of social media on the treatment of acne in Saudi Arabia.

According to the findings, approximately 42.4% of those surveyed utilized social media platforms for acne treatment advice. This high prevalence also appears in previous studies, which found that nearly 45% of individuals reported using social media for acne treatment advice [11]. This finding is in line with an earlier study that showed how patients were influenced by social media in terms of acne treatment (51.9%) [15].

In terms of gender, there was a statistically significant distinction between social media users and nonusers. Women were more susceptible to social media-influenced acne treatment recommendations. Moreover, based on the findings of non-dermatological studies, women proved to be more involved in using the Internet and social media for medical-related queries [16]. This could be due to a better level of health knowledge and active health-seeking behavior [17-19]. Additionally, when the relationship between education level and utilizing social media for advice was investigated, it was shown that 74.1% of people who used social media for advice were educated at the college level, and there was a positive correlation between the two variables. There was also a link between the severity of acne and the use of social media for acne treatment advice. According to Gantenbein et al., women with a greater level of education and patients with a higher dermatological burden were more likely to search for medical information online [20].

The findings of the present study also suggest that many social media users made lifestyle adjustments to manage their acne. The majority chose to increase their water intake and implement dietary changes (40.4% and 37.3%, respectively), although there is limited evidence that a high glycemic index diet and dairy consumption are linked to acne worldwide, especially in Saudi Arabia. This result is further supported by Yousaf et al., who found that many social media users (48%) implemented lifestyle changes that were not recommended by the published guidelines, such as dietary adjustments [11].

Furthermore, based on social media recommendations, 33.7% of social media users began treatment without a prescription. Benzoyl peroxide, retinoid, tretinoin, topical antibiotics, and numerous herbal medicines were among the treatments used, some of which had significant side effects on the patients’ skin. According to research, patients with acne vulgaris looked through social media most frequently for information on over-the-counter treatments (79.31%) [11].

Consequently, these findings should alert healthcare practitioners, because obtaining prescription drugs without a prescription could harm individuals. The extent of public misconception regarding acne treatment suggests the imperativeness of proper patient education for those suffering from acne.

Predictably, only 21.2% of social media users reported considerable improvement in their acne. This could be attributed to the fact that social media content is less accurate than content from other healthcare sources [21]. Studies have shown acne treatment information on Instagram to be unreliable [13]. According to a previous study, nearly half of the participants did not seek advice from a doctor before attempting any social media-related acne treatment, and it took around a month for half of the participants who were inspired by social media to see an improvement in their acne. Of the participants, 15% saw no change whatsoever [15]. In contrast, 32.3% of the participants did not search for acne treatment information on social media and were more willing to pursue medical help.

Finally, Instagram (34%) was the most popular platform among social media users, followed by YouTube (33.2%). This finding was similar to that of a study conducted by Aslan Kayıran et al., which showed that participants primarily utilized Google to find information on acne vulgaris, followed by Instagram and YouTube, and a quarter of them read patient blogs [22]. Hence, since Instagram and YouTube are very popular among young people, it is not surprising that they are the most commonly used platforms for obtaining information [23]. This highlights the importance of learning more about acne patients’ search habits to effectively target medical content from evidence-based sources.

Although our sample size was far larger than that of any previously published work in the field and the study met its objectives, several limitations should be noted. First, we used a questionnaire that has not been checked for reliability and validity in a different patient population. Thus, the results should be evaluated with caution. Second, because the majority of those surveyed were women, the results are likely to underestimate the proportion of male patients who sought acne treatment recommendations through social media.

## Conclusions

Social media has an impact on acne treatment in Saudi Arabia, particularly among women, adolescents, and young adults. Furthermore, the majority of people who sought advice on social media were college graduates; despite their higher level of education, they were more susceptible to social media-influenced acne treatment recommendations.

Additionally, a significant number of patients began treating their acne based on social media advice, some of which are not recommended by established guidelines and have significant side effects on the skin. It is therefore critical for dermatologists to inquire about any acne treatment advice given on social media and address any misconceptions accordingly.

## Appendices

Questionnaire

1) Gender:

o Female

o Male

2) Nationality:

o Saudi

o Non-Saudi

3) City or residence:

o Western region

o Central region

o Eastern region

o Southern region

o Northern region

4) Age (in years):

5) Social status:

o Single

o Married

o Divorced or widowed

6) Level of education

o Uneducated

o Primary school graduate

o Secondary school graduate

o High school graduate

o University graduate

o Master/doctoral degrees

7) Do you have acne now?

o Yes, always

o Yes, sometimes

o No

8) Have you ever had acne in the past?

o Yes

o No

9) If your answer was yes, how bad is/was your acne usually?

o Mild

o Moderate

o Severe

10) What is your first acne treatment approach sources, usually?

o Social media recommendation

o Family/friend

o Medical professional

o Pharmacist

o Other

11) If your answer was social media, what is the platform you prefer to use for acne treatment recommendations? (You can choose more than one answer.)

o WhatsApp

o YouTube

o Facebook

o Snapchat

o Twitter

o Instagram

o Tumblr

o TikTok

12) Have you tried any treatment for acne based on a social media recommendation?

o Yes

o No

13) What is the social media treatment recommendation you use usually? (You can choose more than one answer.)

o Over-the-counter product

o Self-made recipe

o Diet modification

o Exercise modification

o Supplement use

o Drink a lot of water

14) Did you find any changes in your acne?

o No, I didn’t find any change

o Minimal improvement

o Significant improvement

15) Have you experienced any adverse reactions based on the social media recommendations for acne treatment?

o Yes

o No

16) If your answer was yes, please mention it:
